# Heat Shock Protein 60 in Eggs Specifically Induces Tregs and Reduces Liver Immunopathology in Mice with *Schistosomiasis Japonica*

**DOI:** 10.1371/journal.pone.0139133

**Published:** 2015-09-29

**Authors:** Sha Zhou, Xin Jin, Xiaojun Chen, Jifeng Zhu, Zhipeng Xu, Xuefeng Wang, Feng Liu, Wei Hu, Liang Zhou, Chuan Su

**Affiliations:** 1 Department of Pathogen Biology and Immunology, Jiangsu Key Laboratory of Pathogen Biology, Nanjing Medical University, Nanjing, Jiangsu, China; 2 Department of Microbiology and Microbial Engineering, School of Life Science, Fudan University, Shanghai, China; 3 Department of Pathology, Department of Microbiology and Immunology, Northwestern University, Feinberg School of Medicine, Chicago, Illinois, United States of America; Wayne State University School of Medicine, UNITED STATES

## Abstract

**Background:**

Parasitic helminths need to suppress the host immune system to establish chronic infections. Paradoxically, immunosuppression induced by the worm also benefits the host by limiting excessive inflammation and tissue damage, which remains the major cause leading to serious morbidity and mortality. Regulatory T cells (Tregs) are key immune regulators of this mutualism. The successive rise in Tregs during schistosome infection plays a critical role in immunoregulation. We and others previously showed that *Schistosoma japonicum* (*S*. *japonicum*) egg antigens (SEA) induce Tregs both *in vitro* and *in vivo*. In addition, we identified that SjHSP60 derived from SEA significantly induces Tregs *in vivo* and *in vitro*. However, the contribution of SjHSP60 in SEA to Treg induction and the related mechanisms of the Treg induction have not yet been identified.

**Methodology/Principal Findings:**

In this study, we showed that *S*. *japonicum* stress protein HSP60 (SjHSP60) was constitutively and extensively expressed in eggs of *S*. *japonicum*. SjHSP60 specially induced Tregs *in vivo* and *in vitro* without inducing other CD4^+^ T sub-populations including Th1, Th2 and Th17 cells. Furthermore, we showed that the SjHSP60-depleted SEA almost lost the ability *in vitro* and displayed a significant impaired ability to induce Tregs *in vivo*. Finally, our study illustrated that the mechanisms of SjHSP60-mediated induction of Tregs are through both conversion of CD4^+^CD25^-^ T cells into CD4^+^CD25^+^Foxp3^+^ Tregs and expansion of preexisting CD4^+^CD25^+^Foxp3^+^ Tregs in a TLR4-dependent manner.

**Conclusions/Significance:**

Collectively, our findings identify SjHSP60 as a major parasitic contributor of Treg induction in *S*. *japonicum* egg antigens, which not only contributes to the better understanding of the mechanism of immunoregulation during helminth infection, but also suggests its potential as a therapeutic target for control of immunopathology, allergic and autoimmune diseases.

## Introduction

In response to various pathogenic infections (e.g., parasites), the host immune system develops complex signaling networks to eliminate the invading pathogens. Oftentimes, pathogens can evade immune attack by compromising the host immune system to establish a chronic infection. Meanwhile, dampening excessive immune responses is also beneficial for the host to limit tissue immunopathologic damage and survive the acute infection period. This mutualism between the pathogen and the host is one of the most important phenotypic plasticity and fitness consequences of chronic infection [1–5]. Schistosomiasis is one of the most prevalent helminthic diseases of the tropics, chronically affecting an estimated 200 million people worldwide, in which chronic egg-induced granulomatous inflammation in host organs can lead to fibrosis and eventual death [6–8].

Parasitic helminthes, including schistosomes, infections are potent stimuli for the generation of CD4^+^CD25^+^Foxp3^+^ regulatory T cells (Tregs), which remain the most prominent population of immunoregulatory cells and are key regulators of the immunopathology during helminth infections [9–11]. The importance of Tregs has been demonstrated in *S*. *mansoni*-infected mice, where *in vivo* ablation of Tregs resulted in larger egg-induced granulomas [12,13]. Previous reports including our own showed that schistosome egg antigens (SEA) induce Tregs *in vitro* and *in vivo* [14–18]. In addition, we identified that SjHSP60 from SEA significantly induces Tregs *in vivo* and *in vitro* [19]. However, the contribution and the mechanism of SjHSP60 in Treg induction and granulomatous pathology control during schistosome infection have not been demonstrated.

Here, we further show that SjHSP60 is a major contributor in SEA for TLR4-dependent Treg induction, including both specifically *de novo* conversion of non-Tregs into Tregs and expansion of preexisting Tregs, to limit the host liver immunopathologic damage. Our study indicates a potential role of SjHSP60 in controlling immunopathology after *S*. *japonicum* infection, allergic or autoimmune diseases.

## Materials and Methods

### Ethics statement

Animal experiments were performed in strict accordance with the Regulations for the Administration of Affairs Concerning Experimental Animals (1988.11.1), and all efforts were made to minimize suffering. All animal procedures were approved by the Institutional Animal Care and Use Committee (IACUC) of Nanjing Medical University for the use of laboratory animals (Permit Number: NJMU 13–0206).

### Mice

Specific pathogen-free (SPF) 8-wk-old female C57BL/6, C57BL/10, TLR2^-/-^ (C57BL/6) and TLR4^-/-^ (C57BL/10) mice were purchased from the Model Animal Research Center of Nanjing University (Nanjing, China). Animal care and all procedures were carried out in accordance with the guidelines of Chinese animal protection laws and with permission from the Institutional Review Board.

All mice were bred in an SPF animal facility and housed 4 per cage bedded with heat-treated chipped hardwood at the weekly change. The facility uses a 12 h light/dark cycle, and a standardized room temperature (≈ 22°C) and humidity (30–70%). The provided food and water were freely available. Cages were checked daily to monitor mice for signs of morbidity including body weight loss, hunching, lethargy, or anorexia. For the sacrifice, mice at the end of the study were anesthetized by an intraperitoneal injection of xylazine (12mg/kg) and ketamine (80mg/kg), followed by cervical dislocation. There was no mice severely ill and unexpected death prior to the experimental endpoint in this study.

### Preparation of monoclonal antibody (mAb) and immunohistochemistry

Purified SjHSP60 protein cleared of LPS was used as an immunogen to prepare a mAb (clone S-129-5) in female BALB/c mice by the Antibody Research Center of Shanghai Institutes for Biological Sciences (SIBS, China). The affinity and specificity of mAb was evaluated by Western blot detection (S1 Fig) and then the mAb was used in the following experiments.

Paraformaldehyde-fixed *S*. *japonicum* adult worms and eggs were embedded in paraffin and then sectioned (5 μm). After deparaffinization, sections were blocked with 5% bovine serum albumin (BSA; Sigma-Aldrich, St. Louis, MO) and then incubated with SjHSP60 mAb (S-129-5; 1:100 dilution) or isotype-matched control antibody (mouse IgG1; 1:100 dilution; eBioscience, San Diego, CA) for 2 h at 37°C. The sections were then washed and incubated for 1 h at room temperature with secondary antibody in a ratio of 1:500 (HRP-conjugated anti-mouse IgG; Sigma-Aldrich). After washing, sections were developed using diaminobenzidine (DAB; Sigma-Aldrich) and washed under tap water. The staining process was optically controlled and stopped after 3 min by a water passage. Subsequently, sections were then counter stained with hematoxylin and observed under a light microscope (Zeiss, Oberkochen, Germany).

### Preparation of SEA and SWA (soluble worm antigens)

SEA or SWA was prepared as previously described [20,21]. Antigens were sterile filtered and endotoxin removed to <0.01 EU/μg by using Polymyxin B-Agarose (Sigma-Aldrich) and the LAL assay kit (BioWhittaker, Walkersville, MD). Protein concentrations were determined by using the Lowry method (DC Protein Assay Kit, Bio-Rad, Hercules, CA). Equal amounts of SEA and SWA were loaded in Western blot analysis.

### Immunoprecipitation (IP) of SjHSP60 from SEA

IP was performed as described previously [22]. For a single IP, 25 μl anti-SjHSP60 mAb (25 μg) was incubated with 200 μl protein G-agarose suspension (50% solution; pH 8.8; Sigma-Aldrich) at 4°C for 3 h. Then anti-SjHSP60 mAb conjugating protein G-agarose suspension was collected by centrifugation, mixed with SEA (200 μg) and incubated with mixing at 4°C for 3 h. Finally, the protein-G was removed by centrifugation. The depletion of SjHSP60 from SEA was analyzed by Western blot. Protein concentrations were determined by using the DC Protein Assay Kit (Bio-Rad). Equal amounts of SEA and SjHSP60-depleted SEA were loaded.

### Infection of mice with *S*. *japonicum*

C57BL/6 mice were infected percutaneously by exposure of the abdominal skin for 20 min to 12 *S*. *japonicum* cercariae of the Chinese mainland strain from infected snails (*Oncomelania hupensis*) obtained from the Jiangsu Institute of Parasitic Diseases (Wuxi, China).

### Immunization protocol

In each experiment, C57BL/6 mice or *S*. *japonicum*-infected C57BL/6 mice at 6 weeks post-infection were divided randomly into different immunized groups consisting of six mice each group. Immunization was performed as previously described [23]. On day 0, each mouse was subcutaneously (s.c., inguinal region) injected with 100 μl of 1:1 (v/v) mixture of antigens (50 μg SEA, 50 μg SjHSP60-depleted SEA, 20 μg SjHSP60, 20 μg OVA, PBS) and incomplete Freund’s adjuvant (Sigma-Aldrich). Each mouse was injected twice with a 14-day interval. Spleens, lymph nodes, livers or serum samples were collected 10 days after the last injection for further analysis.

### Western blot analysis

Equal amounts of SEA and SjHSP60-depleted SEA or SEA and SWA (80 μg) were loaded in each lane for separation by SDS-PAGE and transferred to a nitrocellulose membrane. After blocking in Tris-buffered saline containing Tween 20 (0.1%) (T-TBS) and milk (5%) at room temperature for 2 h, the membrane was then incubated at 4°C overnight with anti-SjHSP60 mAb (S-129-5; 1:500 diluted). After washing with T-TBS, the membrane was incubated at room temperature for 1 h with HRP-conjugated goat anti-mouse IgG secondary antibody (Cell Signaling Technology, Danvers, MA). In parallel, Coomassie Blue staining was used to confirm that the equal amounts of antigens were used in Western blot analysis.

### *In vivo* depletion of Tregs

Tregs were depleted as described in previously published protocols [24]. *S*. *japonicum*-infected C57BL/6 mice were given 0.2 mg/dose/mouse intraperitoneally (i.p.) of a depleting CD25 mAb (PC61; eBioscience) or isotype antibody (IgG1; eBioscience) at days 7, 14 and 21 after the first injection of SjHSP60. Depletion of CD4^+^CD25^+^ Tregs was confirmed in the spleen, LNs and peripheral blood by flow cytometry (FCM).

### Liver pathology

Formalin-fixed, paraffin-embedded liver tissues from infected mice were sectioned (5 μm) and stained with hematoxylin and eosin (H&E) to determine granuloma areas. For each mouse, sizes of 30 granulomas around individual eggs were quantified with the AxioVision Rel 4.7 Imaging System (Zeiss). Data are expressed in area units. All images were captured at 100× magnification using an Axiovert 200M microscope and analyzed with Axiovision software (Zeiss).

The liver sections were stained with 0.1% Sirius red for semi-quantitative analysis of hepatic fibrosis [25]. Six to eight fields from each slide were randomly obtained with an optical microscope (Zeiss) equipped with a digital camera. The red-stained area per total area and the intensity of fibrosis were measured using Image-Pro Plus software (version 6.0 for Windows; Media Cybernetics, Rockville, MD). A total fibrosis density score was determined by dividing the image intensity by the image area. Intensity exclusion parameters were identical for each of the images captured.

### Cell isolation

Spleens and LNs (axillary, inguinal and mesenteric LNs) were pressed through nylon nets to prepare single-cell suspensions. Cells were washed, counted after lysing the red blood cells and then stained with PerCP-Cy5.5-CD3, FITC-CD4 or APC-CD25 mAb (eBioscience). CD4^+^CD25^-^ T cells and CD4^+^CD25^+^ Tregs were respectively isolated by using a FACSAria cell sorter (BD Biosciences, San Jose, CA). Procedure typically yields 99% enriched cells in all cases as determined by FCM analysis.

Antigen presenting cells (APCs) were prepared from spleen cells by negative selection using CD90.2 magnetic microbeads (Miltenyi Biotec, Bergisch Gladbach, Germany) to deplete T cells as described previously [26,27], and were then irradiated with 30 Gy at 2.7 Gy/min using a ^137^Cs source (Gammacell 1000 Elite; Nordion International, Kanata, ON, Canada).

To isolate CD11b^+^ Mφ and CD11c^+^ DC, mice splenic mononuclear cells were isolated by using Percoll density-gradient centrifugation as described previously [28], then incubated with anti-CD11b (Miltenyi Biotec) or anti-CD11c microbeads (Miltenyi Biotec) and captured on MS columns (Miltenyi Biotec) according to the manufacturer’s instructions as previously described [29]. The purity of the isolated CD11b^+^ Mφ or CD11c^+^ DC was greater than 95%.

### *In vitro* assays to detect the activities of HSP60 on Th cell/Treg induction and Treg conversion/proliferation

To determine the *in vitro* induction of Tregs or Th cells (Th1, Th2, and Th 17 cells), spleen or LN cells were cultured in 96-well round-bottom plates at 5 × 10^**5**^ cells per well in complete RPMI 1640 medium (Gibco, Grand Island, NY). SEA (20 μg/ml), SjHSP60-depleted SEA (20 μg/ml), SjHSP60 (0.2 μg/ml) or OVA (0.2 μg/ml) was added to each culture in quadruplicates for stimulation. After 3 days in culture, cells were collected for FCM analysis.

To investigate Treg conversion induced by SjHSP60 *in vitro*, FACS (fluorescence activated cell sorting) purified CD4^+^CD25^-^ T cells (2 × 10^**5**^ cells/well) were co-cultured with irradiated APCs (1 × 10^**5**^ cells/well), DC (1 × 10^**5**^ cells/well), Mφ (1 × 10^**5**^ cells/well) purified from mice deficient in TLR2, TLR4 or their control littermates, or pre-incubated for 1 h with 20 μg/ml of anti-TLR4 (eBioscience), anti-mouse TLR2 (eBioscience) or their isotype control antibodies (eBioscience) in triplicate in 96-well round-bottom culture plates with stimulation of SjHSP60 (0.2 μg/ml), OVA (0.2 μg/ml) or PBS. After 3 days, cells were collected for FCM analysis.

To investigate Treg proliferation induced by SjHSP60 *in vitro*, total spleen cells or CD4^+^CD25^+^ Tregs purified from mice deficient for TLR2, TLR4 or control littermates, were cultured alone or with 0.5 μg/ml SjHSP60 or 1 μg/ml anti-CD3 mAb (BD Bioscience) as a proliferation control [30,31]. After 3 days, spleen cells were collected and analyzed for Ki-67 expression in CD4^+^CD25^+^ Tregs by FCM. CD4^+^CD25^+^ Tregs were incubated for 3 days and pulsed for the last 16 hours with [^3^H]thymidine (0.5 μCi). Cells were then collected and thymidine incorporation was measured with a liquid scintillation counter.

### Immunofluorescence staining and FCM

To detect Th1, Th2 and Th17 cells, single-cell suspensions of splenocytes from each mouse or from *in vitro* cultures were prepared. Each sample (1×10^6^ cells) was stimulated with 25 ng/ml phorbol myristate acetate (PMA) and 1 μg/ml ionomycin (Sigma-Aldrich) in complete RPMI 1640 medium in the presence of 1 μl/ml Golgistop (BD PharMingen) for 6 h at 37°C in 5% CO_2_. After 6 h, the cells were collected and surface stained with anti-CD3-APC (eBioscience) and anti-CD4-FITC (eBioscience). The cells were washed, fixed, permeabilized with Cytofix/Cytoperm buffer (BD PharMingen) and subsequently intracellularly stained with PE-conjugated antibodies against IFN-γ, IL-4, IL-17A or isotype antibody (eBioscience) as control according to the manufacturer’s protocol and analyzed by FCM using a FACSCalibur instrument (BD Bioscience) and CellQuest software (BD Bioscience). Cells were gated on CD3^+^CD4^+^ T cells.

To evaluate the induction of CD4^+^CD25^+^Foxp3^+^ Tregs, single-cell suspensions from spleens or LNs of immunized mice or from co-cultures were analyzed using the Mouse Regulatory T Cell Staining Kit (eBioscience). A total of 1 × 10^**6**^ cells were stained with FITC-labeled anti-mouse CD4 and APC-labeled anti-mouse CD25. The cells were subsequently permeabilized with cold Fix/Perm Buffer, and Fc receptors of cells were blocked with anti-mouse CD16/32 for 15 min. The PE-labeled anti-mouse Foxp3 or PE-labeled rat IgG2a isotype control antibody was then added based on the manufacturer’s recommendations. Cells were then analyzed by FCM using a FACSCalibur instrument (BD Bioscience) and CellQuest software (BD Bioscience). Cells were gated on CD4^+^ T cells.

To investigate Treg proliferation *in vitro*, total spleen cells were stained with FITC-labeled anti-mouse CD4 (eBioscience), APC-labeled anti-mouse CD25 (eBioscience) and PE-labeled anti-mouse Ki-67 (eBioscience) and then analyzed by FCM using a FACSCalibur (BD Bioscience) and CellQuest software (BD Bioscience). Cells were gated on CD4^+^CD25^+^ Tregs.

### Supplementary methods

For more information, see S1 Text.

### Statistical analysis

Statistical analysis was performed using the SPSS program (version 11.0 for Windows; SPSS, Inc., Chicago, IL). The differences between two groups were analyzed by the Student’s *t* test. The differences between more than two groups were analyzed with one-way analysis of variance (ANOVA) with an LSD post hoc test. *P* values < 0.05 were considered significant.

## Results

### SjHSP60 specifically induces Tregs

**Fig 1:**
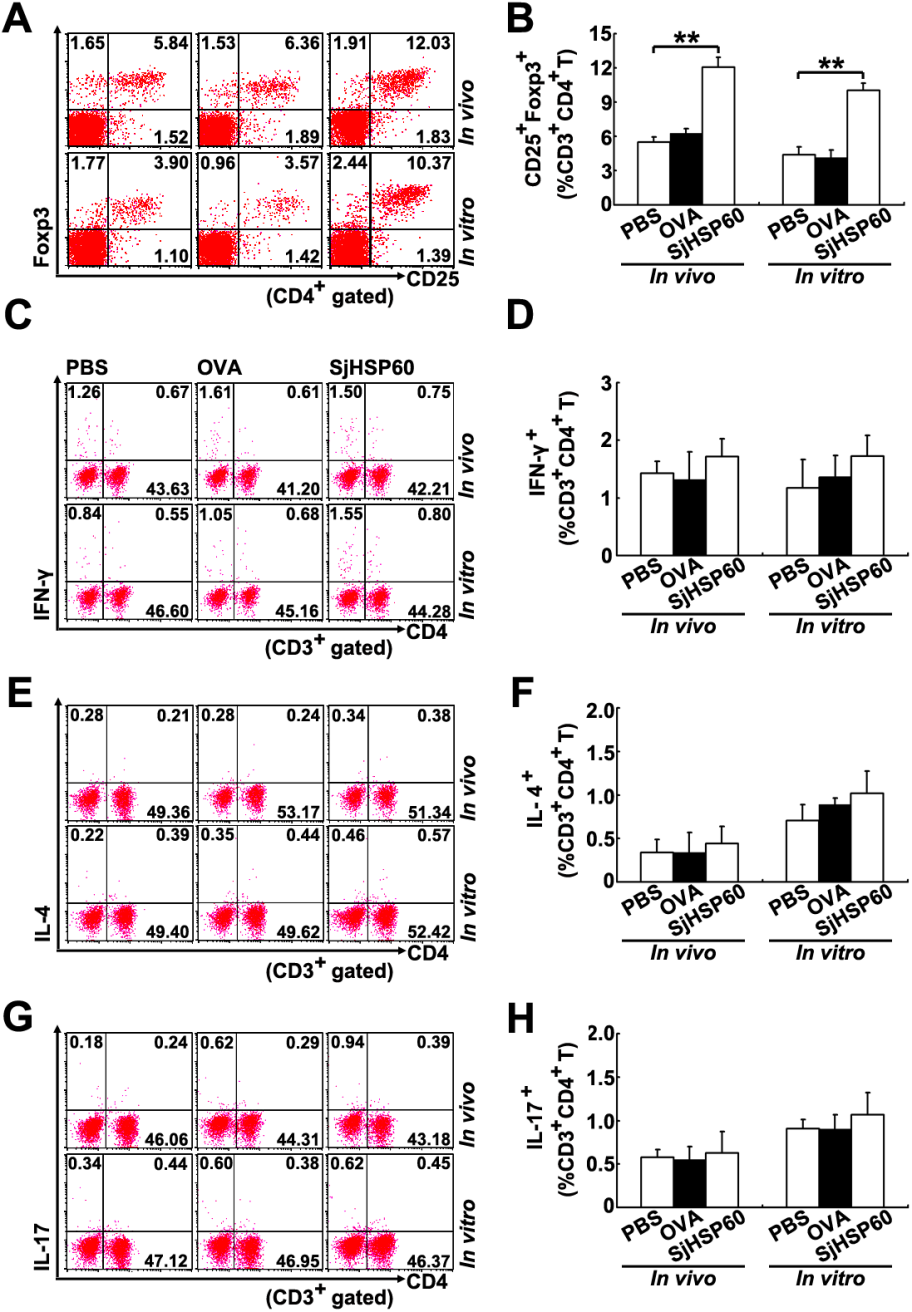
SjHSP60 specifically induces Tregs. For *in vivo* treatment, mice were immunized with SjHSP60 as described in Materials and Methods. Mice were sacrificed 10 d after the last immunization. Single cell suspensions of spleen cells were prepared. For *in vitro* treatment, total spleen cell from normal mice were cultured in the presence or absence of 0.2 μg/ml SjHSP60 or OVA. After 3 days, cells were collected. CD4^+^CD25^+^Foxp3^+^ Tregs **(A),** IFN-γ^+^ Th1 **(C)**, IL-4^+^ Th2 **(E),** or IL-17^+^ Th17 **(G)** cells were identified by FCM. Cells were gated on CD3^+^ or CD4^+^ T cells. FCM plots are representative of one of three independent experiments. Proportions of Tregs **(B)**, Th1 **(D)**, Th2 **(F)**, and Th17 **(H)** cells in CD4^+^ T cells. Bar graphs are shown as the mean ± SD of 15 mice or triplicate cultures from 3 independent experiments. **P < 0.01.

Our previous study showed that SjHSP60 has the ability to induce Tregs *in vivo* and *in vitro* [19]. However, the specificity of SjHSP60-mediated Treg induction is still unknown. Thus, we investigated the induction effects of SjHSP60 on other CD4^+^ T-cell subpopulations (Th1, Th2, and Th17 cells). Results showed that SjHSP60 treatment *in vitro* or *in vivo* induced pronounced increase of Tregs (Fig 1A and 1B), but not Th1, Th2, and Th17 cells (Fig 1C–1H), when compared to PBS or OVA-treated controls. These data suggests that SjHSP60 induces Tregs specifically.

### SjHSP60-depleted SEA showed impaired ability of Treg induction

**Fig 2:**
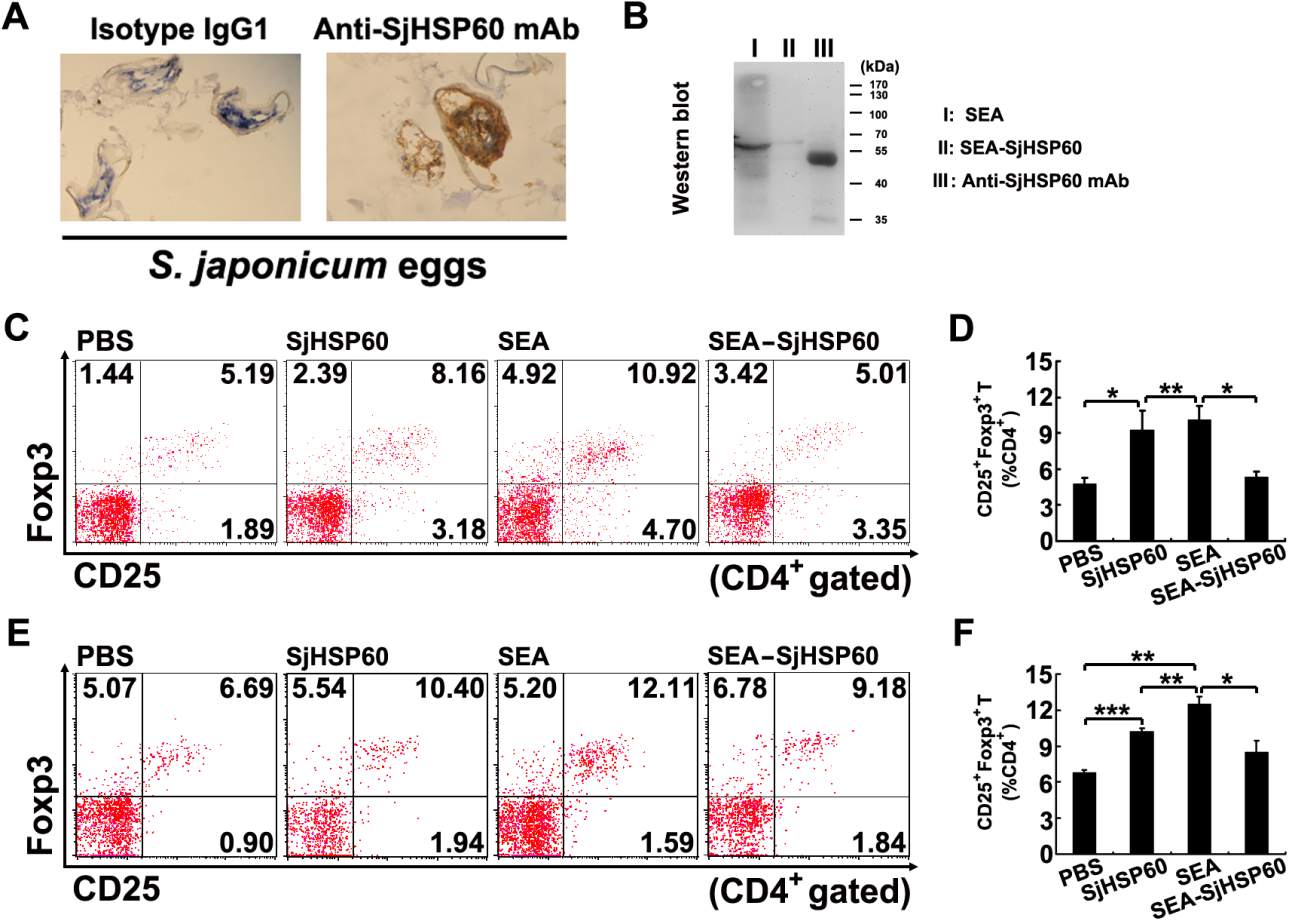
SjHSP60-depleted SEA showed impaired ability of Treg induction. **(A)** Immunohistochemical detection was carried out with the anti-SjHSP60 mAb and isotype-matched antibody. Original magnification: 400×. **(B)** The depletion of SjHSP60 from SEA was proved by Western blot. Equal amounts of SEA and SjHSP60-depleted SEA were loaded. Anti-SjHSP60 mAb (heavy chain ≈ 50 kDa, light chain ≈ 25 kDa) was loaded as a control. Shown is one representative result from three independent experiments. **(C and D)** Total spleen cells from normal mice were cultured in the presence or absence of 0.2 μg/ml SjHSP60, 20 μg/ml SEA or 20 μg/ml SjHSP60-depleted SEA. After 3 days, cells were collected and CD4^+^CD25^+^Foxp3^+^ Tregs were identified by FCM. **(E and F)** Mice were injected with SjHSP60, SEA or SjHSP60-depleted SEA. Ten days after the final injection, CD4^+^CD25^+^Foxp3^+^ Tregs in spleen of individual mice were analyzed by FCM. Five mice were used in each experimental group. FCM plots are representative of one of three independent experiments. Cells were gated on CD4^+^ T cells. All bar graphs indicate average percentages ± SD of CD4^+^CD25^+^Foxp3^+^ Tregs representative of one of three independent experiments. *P < 0.05, **P < 0.01, ***P < 0.001.

We and others previously showed that schistosome egg antigens (SEA) induce Tregs [14–18]. However, the specific and/or dominant antigen(s) in SEA that govern Treg induction have not yet been identified. Immunohistochemistry results showed that SjHSP60 is constitutively and extensively expressed in both eggs and adults of *S*. *japonicum* (Fig 2A and Figure A in S2 Fig). However, Western blot analysis showed that the expression of SjHSP60 in eggs is higher than that in adults (Figure B in S2 Fig). To further determine whether SjHSP60 is a dominant component in SEA for Treg induction, SjHSP60 was depleted from SEA by IP with anti-SjHSP60 mAb. Western blot analysis showed a significant decrease of SjHSP60 in SEA after depletion (Fig 2B). Consistent with previous studies, SEA injection or stimulation of spleen cells *in vitro* resulted in a significantly higher frequency of Tregs (Fig 2C–2F). However, depletion of SjHSP60 from SEA almost completely abrogated SEA-mediated Treg induction *in vitro* (Fig 2C and 2D). In addition, depletion of SjHSP60 from SEA significantly impaired its ability to induce Tregs *in vivo* (Fig 2E and 2F). These data suggests that SjHSP60 is a major contributor for SEA-mediated Treg induction.

### SjHSP60 induces a further increase of Tregs and limits hepatic immunopathology in *S*. *japonicum* infected mice

**Fig 3:**
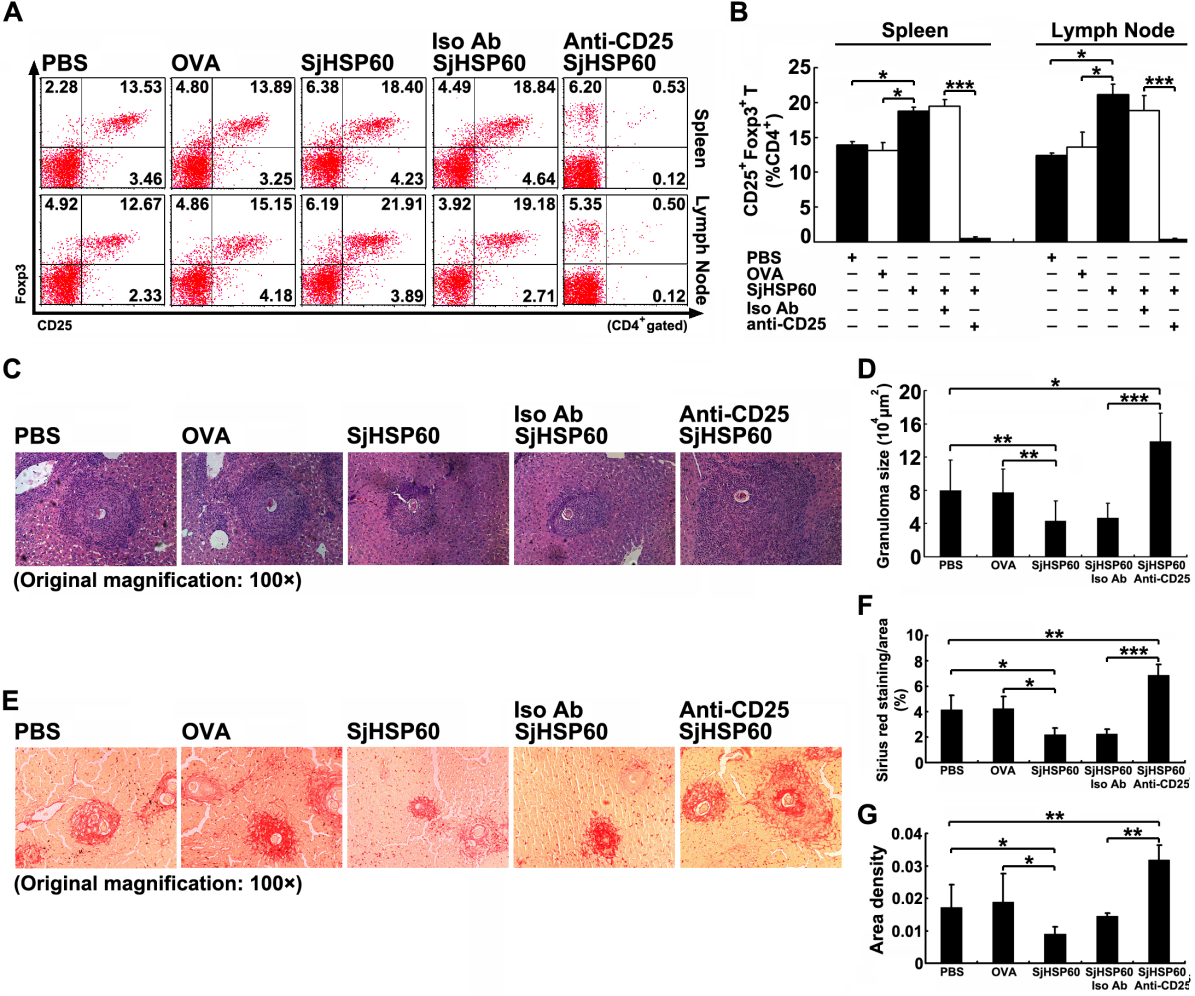
SjHSP60 limits hepatic immunopathology in *S*. *japonicum* infected mice by further increasing Tregs. **(A and B)** Mice were immunized with SjHSP60 and/or injected intraperitoneally with anti-CD25 PC61 depletion mAb to *in vivo* deplete Tregs. Control mice received normal isotype antibody (rat IgG1). Single cell suspension was stained with antibodies against CD4, CD25, and Foxp3 as described in Materials and Methods. CD4^+^CD25^+^Foxp3^+^ Tregs were FCM analyzed and cells were gated on CD4^+^ T cells. FCM plots are representative of three independent experiments. **(C)** Livers sections were H&E stained to reveal granulomas (original magnification, 100×). Images are representative of three independent experiments. **(D)** Granuloma areas were measured. **(E)** Livers sections were stained by Sirius red to reveal granulomas (original magnification, 100×). **(F and G)** Quantification of Sirius red staining was performed by Image-Pro Plus software and represented as percentage of stained-area per total area **(F)** and area density **(G)**. Data are means ± SD of 15 mice from three independent experiments. *P < 0.05, **P < 0.01, ***P < 0.001.

Previous studies have shown that Treg depletion increases liver granulomatous responses in mice with schistosomiasis [12,13]. Thus, we studied the ability of SjHSP60 in restriction of hepatic immunopathology in *S*. *japonicum* infected mice. The injection of the *S*. *japonicum*-infected C57BL/6 mice with SjHSP60 resulted in a further increase of Tregs (Fig 3A and 3B). Histological sections stained with hematoxylin-eosin were used to evaluate granulomatous responses caused by eggs in the liver. Significantly decreased granulomatous responses, characterized by smaller mean area of isolated hepatic granuloma, were observed in the livers of SjHSP60-injected mice compared with mice injected with OVA or PBS (Fig 3C and 3D). However, depletion of CD4^+^CD25^+^ Tregs in SjHSP60-injected mice significantly enhanced the formation of hepatic granulomas (Fig 3A–3D). In addition, the severity of liver fibrosis and injury in SjHSP60-injected mice were significantly reduced than those in OVA-injected mice. However, depletion of CD4^+^CD25^+^ Tregs resulted in enhanced fibrosis and injury in SjHSP60-injected mice livers (Fig 3E–3G and S3 Fig). These data indicates that Tregs are critical in the limitation of the *S*. *japonicum* infection-resulted hepatic immunopathology and SjHSP60 contributes to the restriction of hepatic immunopathology by induction of Tregs.

### SjHSP60 increases Tregs by both conversion of CD4^+^CD25^-^ T cells into CD4^+^CD25^+^Foxp3^+^ Tregs and direct expansion of preexisting Tregs

**Fig 4:**
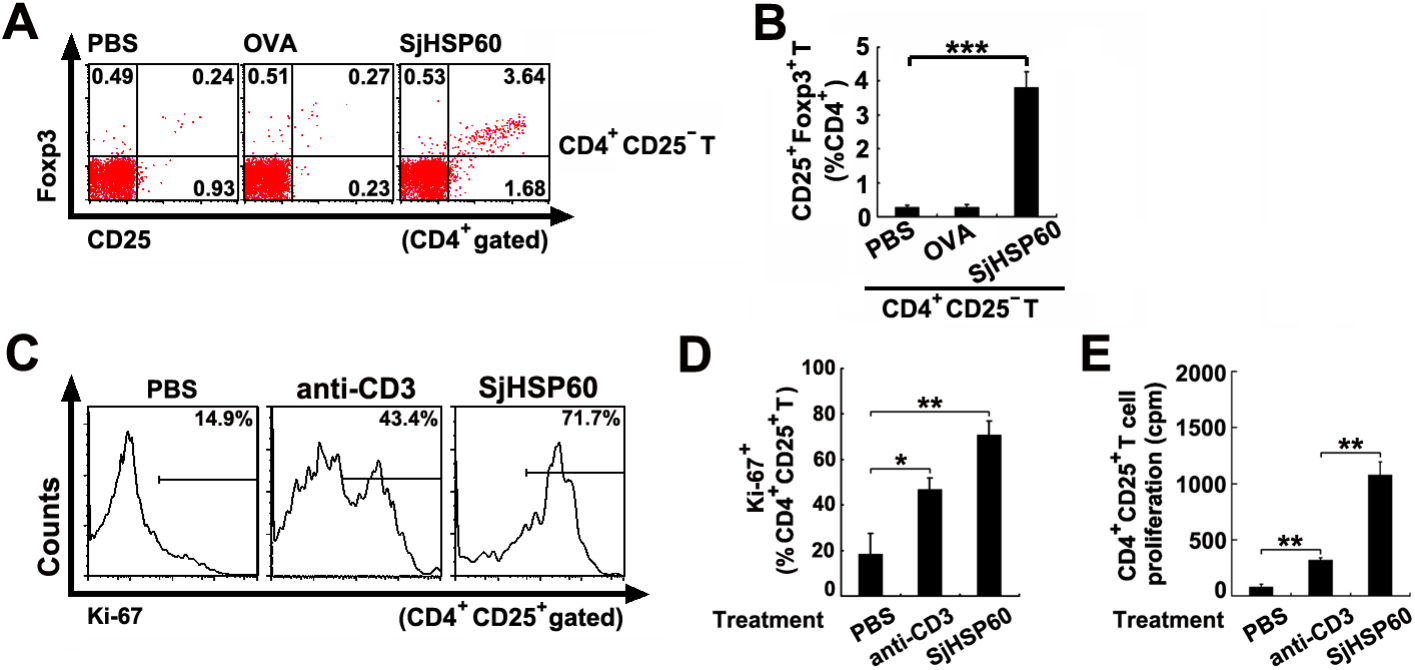
SjHSP60 increases Tregs by both conversion of CD4^+^CD25^-^ T cells into CD4^+^CD25^+^Foxp3^+^ Tregs and direct expansion of preexisting Tregs. **(A and B)** FACS purified CD4^+^CD25^-^ T cells were incubated alone or with SjHSP60, OVA or PBS in the presence of APCs at a 2:1 ratio of T cell:APC. After 3 days, percentages of CD4^+^CD25^+^Foxp3^+^ Tregs were determined by FCM. **(C and D)** Total spleen cells were prepared from normal mice and cultured for 3 days in the presence of 0.5 μg/ml SjHSP60, 1 μg/ml anti-CD3 or PBS. After 3 days, cells were collected and analyzed for CD4, CD25 and Ki-67 by FCM. Cells were gated on CD4^+^CD25^+^ Tregs. **(E)** Purified CD4^+^CD25^+^ Tregs were maintained for 3 days in the presence of 0.5 μg/ml SjHSP60, 1 μg/ml anti-CD3 or PBS. Proliferation of CD4^+^CD25^+^ Tregs were determined by [^3^H]thymidine incorporation. Each assay has been performed three times on independent cell samples and with similar results. Data are the mean ± SD and are representative of three independent experiments. *P < 0.05, **P < 0.01, ***P < 0.001.

Tregs induced peripherally are either converted from CD4^+^CD25^-^ T cells or directly expanded from preexisting Tregs [32–34]. First, we assess whether the increase of Tregs by SjHSP60 could be due to *de novo* Treg conversion. FACS purified CD4^+^CD25^-^ T cells were stimulated with SjHSP60, OVA or PBS for 3 days in the presence of APCs sorted with magnetic microbeads. FCM data showed that SjHSP60 induced a significant conversion of CD4^+^CD25^-^ T cells into CD4^+^CD25^+^Foxp3^+^ Tregs compared to the OVA and PBS control groups (Fig 4A and 4B).

Next, we test whether proliferation of preexisting Tregs also contributed to the SjHSP60-mediated increase of Tregs. Spleen cells were cultured with the stimulation of SjHSP60, anti-CD3 or PBS and assessed the expression of the proliferation marker Ki-67 in Tregs. Result showed that after 3 days, SjHSP60 induced a higher frequency of Ki-67^+^ cells within CD4^+^CD25^+^ Tregs than that anti-CD3 did (Fig 4C and 4D). Furthermore, we cultured FACS purified CD4^+^CD25^+^ Tregs with the same stimulations and measured the incorporation of [^3^H]thymidine. Result in Fig 4E showed that stimulation with anti-CD3 alone induced mild proliferation of Tregs when compared to PBS control without stimulation. However, SjHSP60 stimulation alone induced much higher proliferation of Tregs than that anti-CD3 did (Fig 4E), which directly proved that SjHSP60 promotes a proliferation in preexisting Tregs.

### TLR4 signaling is necessary for SjHSP60-mediated Treg increase

**Fig 5:**
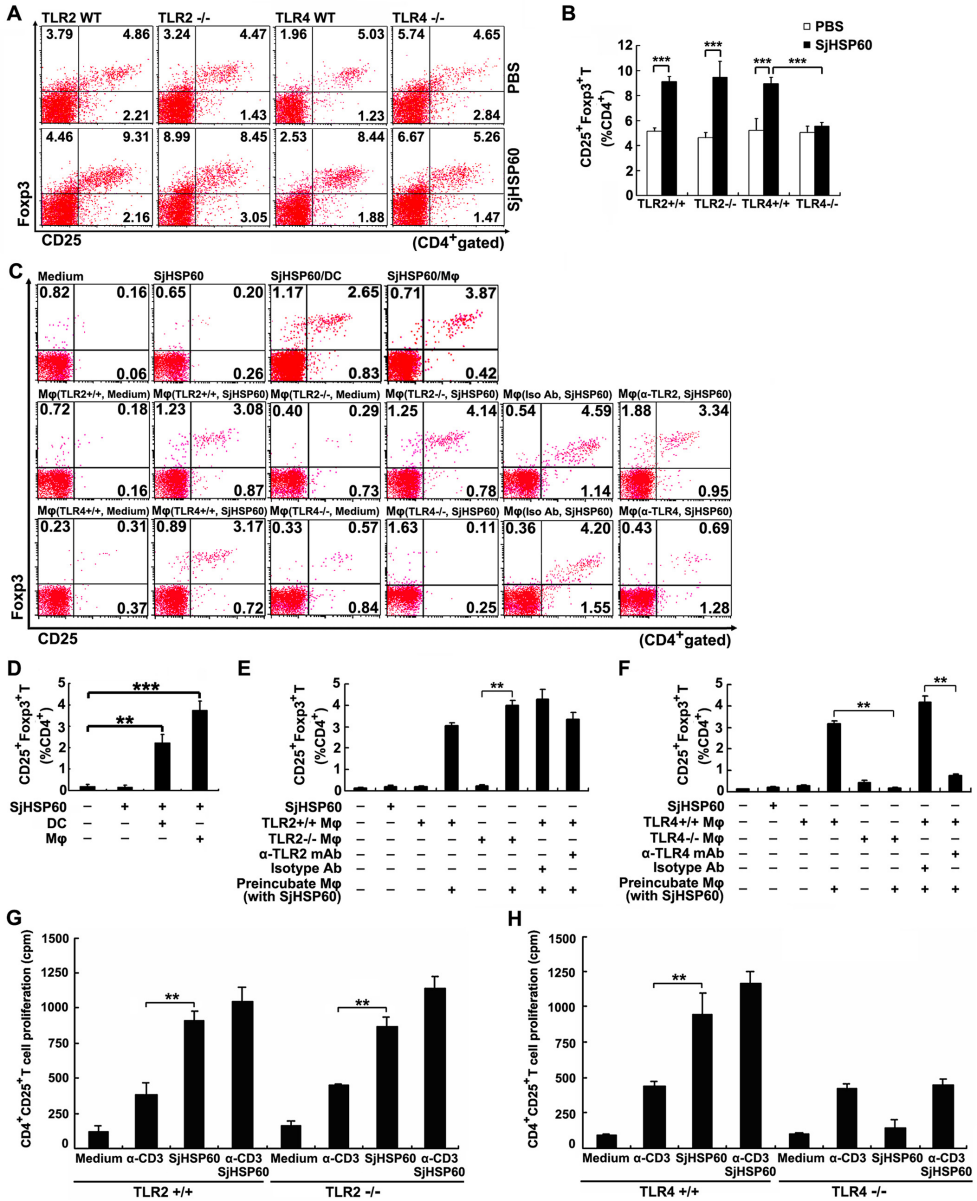
TLR4 signaling is necessary for SjHSP60-mediated Treg increase. **(A and B)** TLR2-/- or TLR4-/- mice or their WT control littermates were immunized with SjHSP60 or PBS. Ten days after the final injection, CD4^+^CD25^+^Foxp3^+^ Tregs in spleen of individual mice were analyzed by FCM. **(C)** Purified CD4^+^CD25^-^ T cells were co-cultured with purified APCs (DC, Mφ, TLR2-/- Mφ, TLR4-/- Mφ, or Mφ pre-treated with anti-TLR2, anti-TLR4 or their isotype control antibodies) at a 2:1 ratio of T cell:APC (DC or Mφ) with or without stimulation of SjHSP60 (0.2 μg/ml). After 3 days, Tregs in co-cultures were analyzed by FCM. **(D-F)** Graphs of CD4^+^CD25^+^Foxp3^+^ Treg frequencies. **(G and H)** CD4^+^CD25^+^ Tregs purified from mice deficient for TLR2, TLR4 or their control littermates were cultured in medium alone or stimulated with 1 μg/ml anti-CD3 and/or 0.5 μg/ml SjHSP60. On day 3, proliferation of CD4^+^CD25^+^ Tregs was determined by [^3^H]thymidine incorporation. FCM plots are representative of one of three independent experiments. Cells were gated on CD4^+^ T cells. The bar graph of average percentages ± SD of CD4^+^CD25^+^Foxp3^+^ Tregs are from 5 mice or triplicate cultures representative of one of three independent experiments. **P < 0.01, ***P < 0.001.

Studies have demonstrated that HSP60 is a natural ligand for TLR2 and TLR4 [35]. To further investigate whether TLR2 and/or TLR4 are involved in SjHSP60-mediated Treg increase, we injected TLR2-/-, TLR4-/- or WT mice with SjHSP60 or PBS and then examined Treg frequency. FCM analysis showed that Tregs were significantly increased in TLR2-/- and WT mice, but not in TLR4-/- mice (Fig 5A and 5B).

*In vitro* results showed that SjHSP60 failed to induce the conversion of CD4^+^CD25^-^ T cells into Tregs in the absence of APCs, e.g. Mφ or DC (Fig 5C and 5D). To further probe the molecular mechanism underlying the SjHSP60-mediated Treg conversion *in vitro*, TLR2 or TLR4-deficient Mφ or anti-TLR2 or anti-TLR4 blocking antibodies were used. As shown in Fig 5C, 5E and 5F, FCM analysis showed that lack of TLR4 signaling on SjHSP60-stimulated Mφ substantially impaired the conversion of CD4^+^CD25^-^ T cells into Tregs *in vitro*. These results suggested that conversion of non-Tregs into Tregs by APCs in response to SjHSP60 stimulation is TLR4-dependent.

In addition, we speculated that SjHSP60-mediated Treg expansion might be the direct effect of triggering of TLR4 on Tregs. FACS purified CD4^+^CD25^+^ Tregs from mice deficient for TLR2 or TLR4 or their wild-type mice were cultured with the stimulation of SjHSP60 and/or anti-CD3. Consistently, CD4^+^CD25^+^ Tregs deficient for TLR4 rather than TLR2 proliferated poorly in response to SjHSP60 stimulation, in contrast to the remarkable proliferation of CD4^+^CD25^+^ Tregs from wild-type mice (Fig 5G and 5H). Taken together, these results suggested that the expansion of preexisting Tregs in response to SjHSP60 stimulation is TLR4-dependent.

### SJMHE1 motif is not necessary in Treg induction by SjHSP60

**Fig 6:**
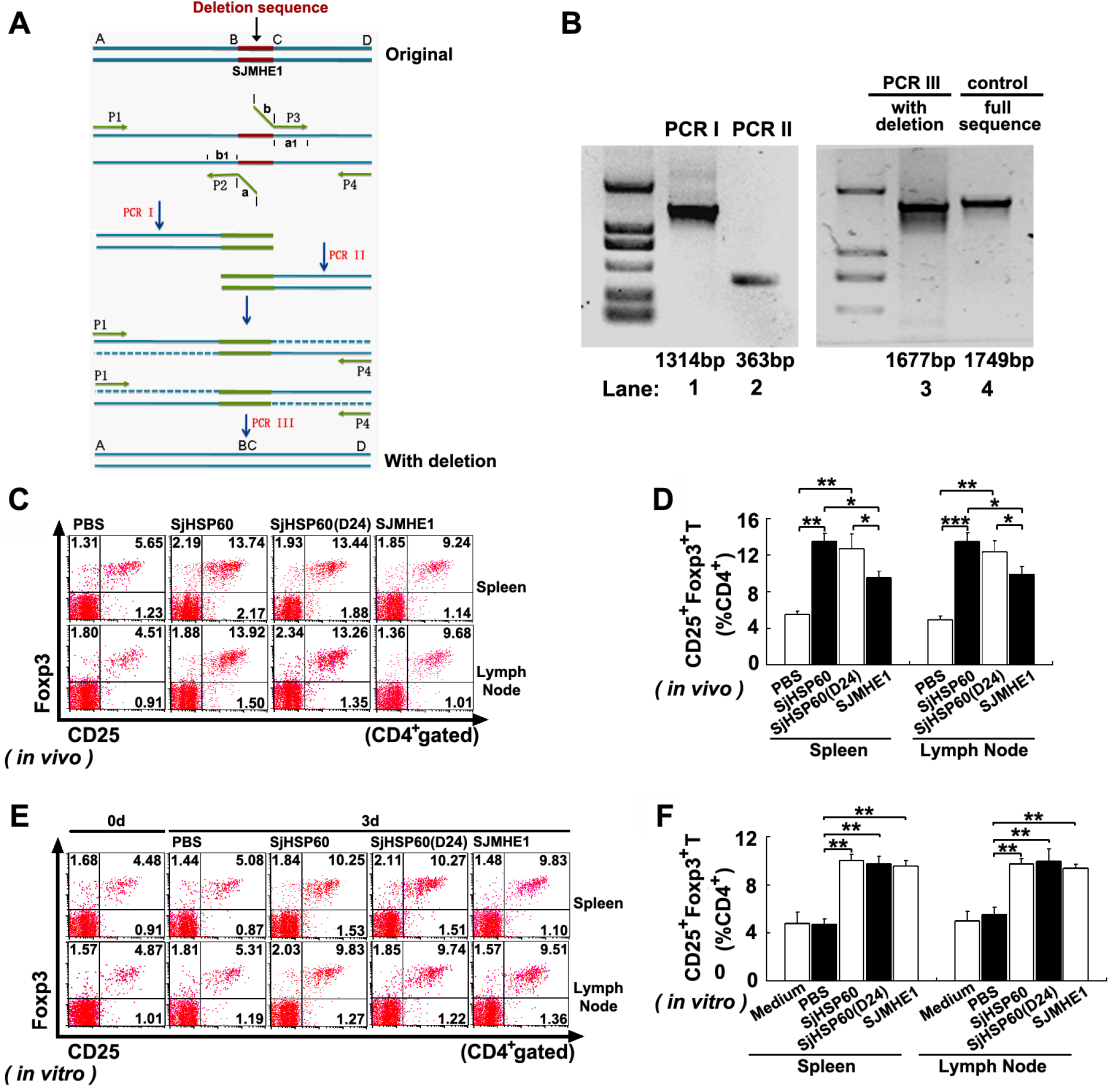
SJMHE1 motif is not necessary in Treg induction by SjHSP60. **(A)** Schematic of PCR design. SJMHE1 motif was deleted from original sequence of SjHSP60 (pGEX-SjHSP60) by PCRs in three steps (I, II and III) with four PCR primers (p1-p4) as described in Supporting Information. P1 and p2, p3 and p4, and p1 and p2 were used in PCR I, II, and III, respectively. Sequences “a” of p2 sequence and “b” of p3 were complementary respectively to the sequences of “a1” and “b1” regions (at right/left of deletion motif), respectively. pGEX-SjHSP60 was used as template in PCR I and PCR II. Mixed PCR1 and PCR2 products were used as template in PCR III. Finally, PCR III product was SjHSP60 sequence with deletion of SJMHE1 motif. **(B)** Lanes 1 and 2 indicate PCR products of PCR I and II respectively. Fragment of PCR I product contained a 44 bp sequence on its 3’-end that overlapped with the 5’-end of fragment of PCR II product. The products of PCR I and II were combined by recombinant PCR with p1 and p4 primers. Deletion of SJMHE1 was identified on the basis of the change in size of PCR III product shown as Lane 3. SjHSP60 with deletion of SJMHE1 was nominated for SjHSP60(D24). Lane 4 is a control with full sequence of SjHSP60. Preparation of recombinant SjHSP60(D24) was the same as that of SjHSP60 described above. **(C and D)** Ten days after the last immunization, spleen or LN cells from PBS, SjHSP60, SjHSP60-D24 or SJMHE1 immunized mice (six mice per group) were analyzed for the percentage of CD4^+^CD25^+^Foxp3^+^ Tregs by FCM. (**E and F**) Pooled spleen and LN cells from normal mice were cultured in medium and stimulated with PBS, SjHSP60, SjHSP60-D24 or SJMHE. After 3 days, CD4^+^CD25^+^Foxp3^+^ Tregs were detected by FCM. All FCM plots are representative of three independent experiments and gated on CD4^+^ T cells. All bar graphs indicate the average percentages ± SD of CD4^+^CD25^+^Foxp3^+^ Tregs of 18 mice from three independent experiments. *P < 0.05, **P < 0.01, ***P < 0.001.

Our previous study demonstrated that a synthetic peptide SJMHE1, which is homologous to amino acid 437–460 of SjHSP60, induced Tregs via TLR2 rather than TLR4 [19]. We then investigated whether SJMHE1 motif is necessary for this function. A truncated form with SJMHE1 motif deleted was generated by PCRs in three steps (Fig 6A and 6B), and it was denoted as SjHSP60(D24). FCM data showed that SjHSP60(D24) and full-length SjHSP60 induced equally high frequency of Tregs *in vivo* and *in vitro* (Fig 6C–6F). These data indicate that SJMHE1 motif is not necessary in Treg induction by SjHSP60, suggesting that other motif(s) in SjHSP60 may be involved in the induction process. Further prediction of the three-dimensional (3D) structure of SjHSP60 revealed a series of potential protein-protein interaction sites of SjHSP60, besides SJMHE1 motif (S4 Fig). Taken together, these results suggest that other predicted interaction site(s) rather than SJMHE1 motif may be responsible for Treg induction by SjHSP60.

## Discussion

Induction of immunosuppressive Tregs has been shown to be one of the most important modes of action in limiting immunopathologic damages in important host organs [9–11,36]. In host with schistosomiasis japonica and mansoni, parasite eggs have been shown to induce Tregs to restrain the development of granulomatous pathology [9,12,37]. However, the specific antigens in eggs that govern Treg induction have not yet been identified.

Our previous findings indicated that SjHSP60, a schistosomal stress protein highly expressed by parasites in response to stresses in the host, is a strong inducer of Tregs *in vitro* and *in vivo* [19]. In this study, we showed that SjHSP60 is constitutively and extensively expressed in eggs of *S*. *japonicum*, which induces Tregs efficiently and specifically. However, the extent to which the Treg-induction capacity of SEA can be attributed to SjHSP60 remains elusive. Considering depletion of SjHSP60 from SEA resulted in significantly decreased induction of Tregs *in vivo* and *in vitro*, we for the first time illustrated that SjHSP60 is a major contributor for SEA-mediated Treg induction. Therefore, although schistosome eggs promote liver immunopathology, specific antigen SjHSP60 present in eggs plays a critical role in controlling excessive immunopathology. However, depletion of SjHSP60 from SEA did not completely impair its ability to induce Tregs *in vivo*, suggesting that there may be some other unknown factors in SEA are able to compensate for SjHSP60 to induce Tregs *in vivo*.

It is well known that *Chlamydia pneumoniae* HSP60 induces a clear Th1 orientation of immune response, representing a crucial component of atherosclerosis pathogenesis [38]. In contrast, our present studies proved that schistosome-derived HSP60 only specially induces Tregs without inducing any other CD4^+^ T sub-populations including Th1, Th2, and Th17 cells, and promotes to reduce pathological reaction. This discrepancy may be explained by these HSP60s derived from different species differ in amino acid sequence and structure, although HSP60 is evolutionarily highly conserved. The mechanisms involved in these differences remain to be determined.

Many studies have reported that Tregs protect against exacerbated schistosome egg-induced immunopathology in important organs in hosts with schistosomiasis [9,12,37]. In this study, injection of SjHSP60 resulted in a further increase of Tregs and decreased hepatic granulomatous damage in *S*. *japonicum*-infected mice. The depletion of Tregs abolished protection against liver immunopathology. Therefore, we speculate that SjHSP60 could be one of important participants which induces Tregs and contributes the control of immunopathology in host with schistosomiasis.

TLR2 and/or TLR4 are natural receptors for HSP60. Activation of TLR2/4 signaling usually promotes the production of pro-inflammatory mediators to induce adaptive antimicrobial effector functions. However, our current dataset suggest that a different outcome of TLR4 signaling upon SjHSP60 stimulation involve promotion of Treg differentiation/expansion, and hence immunosuppression. The exact influence for specific TLR ligands on enhancing or suppressing immune responses is somewhat controversial, which may be due to different immune contexts. Thus, our study suggests that *S*. *japonicum* may “hijack” the host TLR4 signaling pathway to generate an immunocompromised environment for the parasite to sustain chronic infection.

We previously demonstrated that SJMHE1, a synthetic peptide homologous to residues 437–460 of SjHSP60, induced Tregs *in vitro* and *in vivo* only by conversion of non-Tregs into Tregs [19]. However, our results in this study showed that SjHSP60 induces Tregs by both conversion of non-Tregs into Tregs and expansion of preexisting Tregs. Furthermore, our study showed that SJMHE1 motif is not necessary in Treg induction by SjHSP60. And more importantly, SjHSP60 induced Tregs via TLR4 but SJMHE1 via TLR2. Our prediction raises the possibility that there may be many other potential interaction sites that located beyond the region of SJMHE1 motif in SjHSP60 and may have higher affinity and contribute to Treg induction by SjHSP60. However, we cannot exclude another possibility that SJMHE1 motif in SjHSP60 may actually lose the ability to induce Tregs via TLR2, possibly due to conformational difference with peptide SJMHE1 or inaccessibility of surface to other interacting proteins. All of these hypothesis need further study.

Together, we demonstrated for the first time that SjHSP60, as a principal factor for Treg induction in SEA, specifically drives both *de novo* induction and direct expansion of preexisting Tregs in a TLR4 dependent manner to ameliorate hepatic immunopathology during *S*. *japonicum* infection. Moreover, our findings not only pave the way for the use of a defined molecule, SjHSP60, to further delineate the cellular and molecular mechanisms that induce Tregs, but also have important therapeutic implications in exploitation of molecules derived from parasites for allergy, autoimmune diseases and graft-rejection during transplantation.

## Supporting Information

S1 Fig Evaluation of specificity and sensitivity of mAb in Western blot detection. SjHSP60 or SEA was separated by Tris-Tricine SDA-PAGE, blotted onto nitrocellulose membrane, and then stained with mAb (clone S-129-5) against SjHSP60.
(TIF)

S2 Fig Analysis of SjHSP60 expression. **(A)** Immunohistochemical detection of SjHSP60 in adults of *S*. *japonicum* was carried out with the anti-SjHSP60 mAb and isotype-matched control antibody. Original magnification: 100 ×. **(B)** The expression of SjHSP60 in SEA and SWA was analyzed by Western blot. Equal amounts of SEA and SWA were loaded. Shown is one representative result from three independent experiments.
(TIF)

S3 Fig Levels of serum AST/ALT. *S*. *japonicum*-infected mice were immunized with SjHSP60 and/or injected intraperitoneally with anti-CD25 PC61 depletion mAb to *in vivo* deplete Tregs. Control mice received normal isotype antibody (rat IgG1). Serum samples were collected 10 days after the last injection and levels of serum AST/ALT were determined. Data are means ± SD of 15 mice from three independent experiments. *P < 0.05, ***P < 0.001.
(TIF)

S4 Fig Prediction results of 3D structure and protein-protein interaction sites of SjHSP60. Predicted protein-protein interaction sites were mapped onto the surface of the 3D structure of SjHSP60. Amino acids predicted to be interaction sites are shown in red, while non-interacting residues are shown in white.
(TIF)

S1 Text Supporting text. This file contains supplementary methods, including expression and purification of SjHSP60, deletion of SJMHE1 from SjHSP60, evaluation of liver injury, and prediction of protein structure and protein-protein interaction sites.
(DOC)

## Abbreviations

APCs: antigen presenting cells
FACS: fluorescence activated cell sorting
FCM: flow cytometry
LPS: lipopolysaccharide
OVA: ovalbumin
PBS: phosphate buffer solution
SEA: soluble egg antigens
*S. japonicum*: *Schistosoma japonicum*
SjHSP60: *Schistosoma japonicum* stress protein HSP60
SWA: soluble worm antigens
TLR: Toll-like receptor
Tregs: regulatory T cells
