# Supplementary figures and images for: Heat Shock Protein 60 in Eggs Specifically Induces Tregs and Reduces Liver Immunopathology in Mice with Schistosomiasis Japonica
Source: PLoS One. 2015 Sep 29;10(9):e0139133. doi: 10.1371/journal.pone.0139133 (PMC4587937; doi:10.1371/journal.pone.0139133)

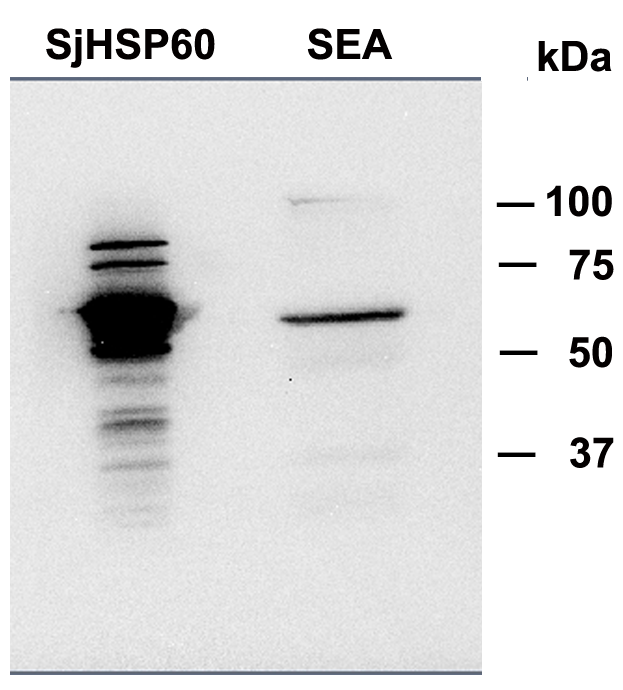

Supplement: S1 Fig — SjHSP60 or SEA was separated by Tris-Tricine SDA-PAGE, blotted onto nitrocellulose membrane, and then stained with mAb (clone S-129-5) against SjHSP60. (TIF) [file pone.0139133.s001.tif]

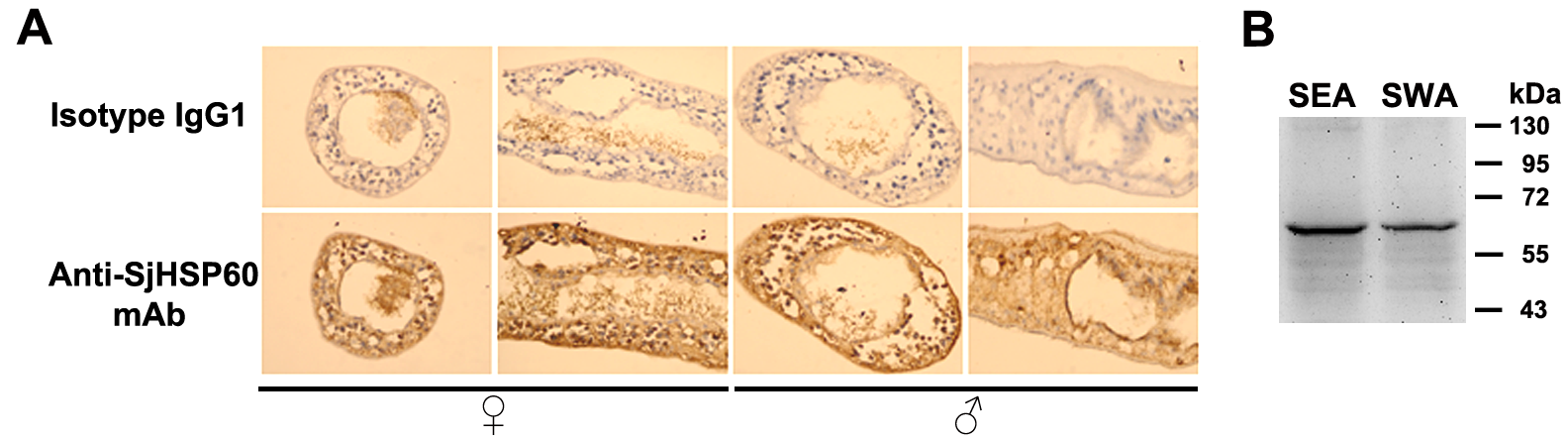

Supplement: S2 Fig — (A) Immunohistochemical detection of SjHSP60 in adults of S. japonicum was carried out with the anti-SjHSP60 mAb and isotype-matched control antibody. Original magnification: 100 ×. (B) The expression of SjHSP60 in SEA and SWA was analyzed by Western blot. Equal amounts of SEA and SWA were loaded. Shown is one representative result from three independent experiments. (TIF) [file pone.0139133.s002.tif]

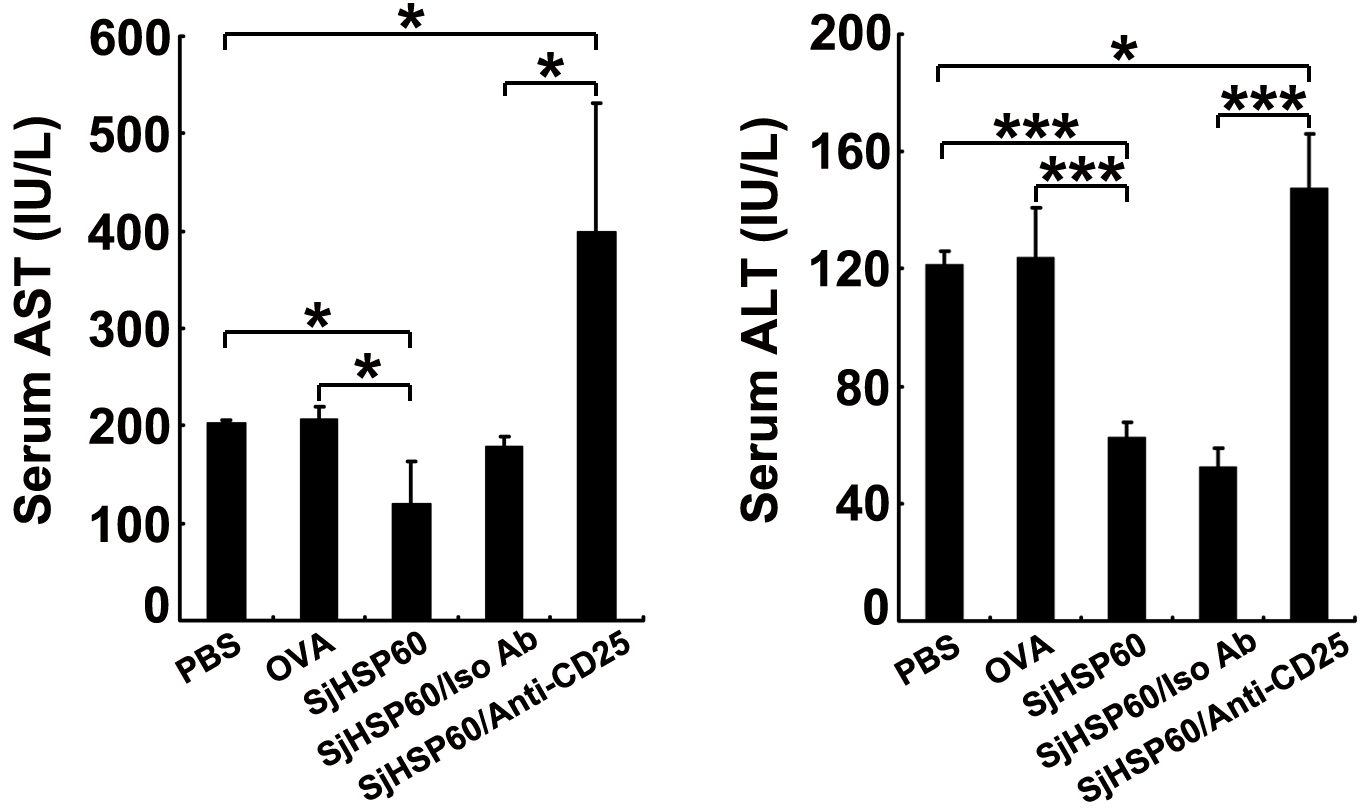

Supplement: S3 Fig — S. japonicum-infected mice were immunized with SjHSP60 and/or injected intraperitoneally with anti-CD25 PC61 depletion mAb to in vivo deplete Tregs. Control mice received normal isotype antibody (rat IgG1). Serum samples were collected 10 days after the last injection and levels of serum AST/ALT were determined. Data are means ± SD of 15 mice from three independent experiments. *P < 0.05, ***P < 0.001. (TIF) [file pone.0139133.s003.tif]

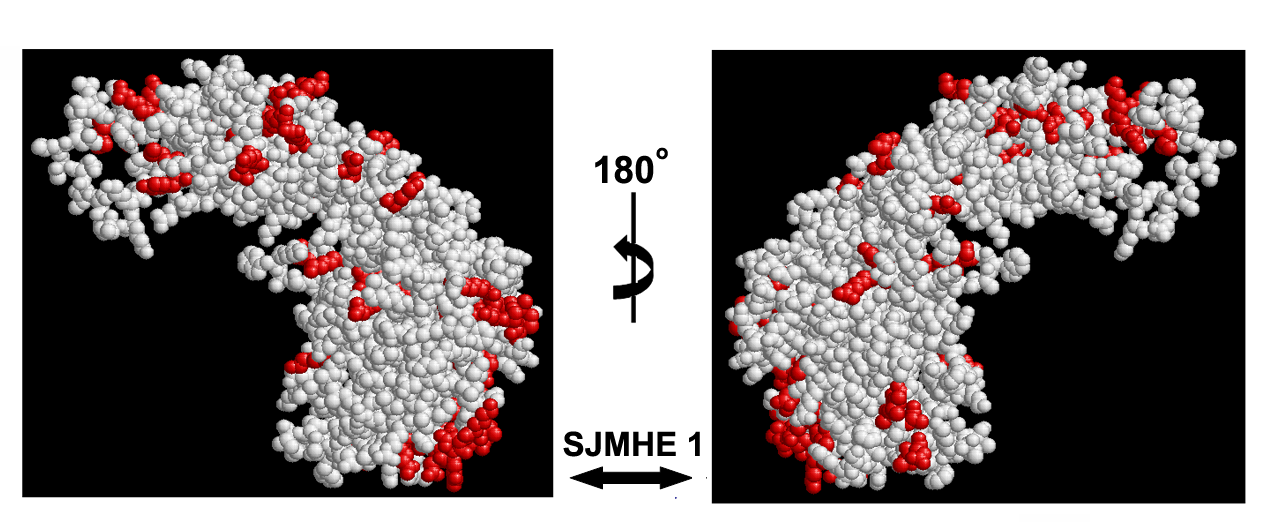

Supplement: S4 Fig — Predicted protein-protein interaction sites were mapped onto the surface of the 3D structure of SjHSP60. Amino acids predicted to be interaction sites are shown in red, while non-interacting residues are shown in white. (TIF) [file pone.0139133.s004.tif]
