# Supplementary material for: Heat Shock Protein 60 in Eggs Specifically Induces Tregs and Reduces Liver Immunopathology in Mice with Schistosomiasis Japonica
Source: PLoS One. 2015 Sep 29;10(9):e0139133. doi: 10.1371/journal.pone.0139133 (PMC4587937; doi:10.1371/journal.pone.0139133)
Supplement: S1 Text — This file contains supplementary methods, including expression and purification of SjHSP60, deletion of SJMHE1 from SjHSP60, evaluation of liver injury, and prediction of protein structure and protein-protein interaction sites. (DOC) [file pone.0139133.s005.doc]

**Supporting Information**

**Supplementary materials and methods**

**Expression and purification of SjHSP60**

The pBluescript-SKII-SjHSP60 plasmid containing the full-length cDNA of SjHSP60 was previously constructed and kindly provided by Wei Hu . The SjHSP60 coding region of approximately 1.7 kb was PCR amplified using a 5’ end primer (5’-cgcggatcccaaccggtgacaatgttacgag-3’) with a *Bam*HI site and a 3’ end primer (5’-ccgctcgagattaagagcaggcagtgtttac-3’) with an *Xho*I site. The full-length HSP60 coding region was cloned into the pGEX-6P-1 expression vector (Amersham Bioscience, Piscataway, NJ) at the *Bam*HI and *Xho*I restriction sites and then transformed into the *Escherichia coli* strain BL21 (Novagen, Madison, WI). After confirmation by DNA sequencing, expression of the GST-SjHSP60 fusion protein was induced with 0.1 mM isopropyl-1-thio-β-D-galactopyranoside (IPTG) for 5 h at 37°C and purified by glutathione-Sepharose 4B (GE Healthcare, Piscataway, NJ) affinity chromatography. Recombinant SjHSP60 was then released by cleaving with PreScission Protease (GE Healthcare) according to the manufacturer’s specifications. Purity of the recombinant SjHSP60 (>95%) was confirmed by sodium dodecyl sulfate-polyacrylamide gel electrophoresis (SDS-PAGE) followed by Coomassie Blue staining. In addition, Polymyxin B-Agarose (Sigma-Aldrich, St. Louis, MO) was used to remove lipopolysaccharide (LPS) and LPS-associated molecules in recombinant SjHSP60 preparations. The endotoxin activity (<0.01 EU/μg, 1 pg/μg) was determined by using the LAL assay kit (BioWhittaker, Walkersville, MD) according to the manufacturer’s instructions.

**Deletion of SJMHE1 from SjHSP60**

To delete the SJMHE1 sequence (amino acids 435-458) in SjHSP60 to generate SjHSP60-D24, the following four primers were used: p1, 5’- cgcggatcccaaccggtgacaatgttacgag-3’; p2, 5’-atccgcacacctgttcgctgtatgccttcctcaattgctgctct-3’; p3, 5’-cagcaattgaggaaggcatacagcgaacaggtgtgcggattgtt-3’; p4, 5’- ccgctcgagattaagagcaggcagtgtttac-3’. P1 and p4 primers were used to amplify the full SjHSP60 sequence described above. Primers p2 and p3 were designed to form the overlapping ends of adjacent PCR products without the SJMHE1 sequence. The PCR1 product was generated by using p1 and p2 primers and pGEX-SjHSP60 as template, and the PCR2 product was produced by using p3 and p4 primers and pGEX-SjHSP60 as template. The PCR1 and PCR2 products were gel-purified using the QIAquick Gel Extraction Kit (Qiagen, Valencia, CA), mixed and used as templates to generate the recombinant PCR3 product encoding SjHSP60 (D24) with p1 and p4 primers. PCRs were run under the following conditions: pre-heating of the reaction mixture for 5 min at 95°C; followed by 32 cycles of denaturation for 1 min at 95°C, primer hybridization for 1 min at 56°C, and DNA synthesis for 4 min or 90 s at 72°C depending on the length of PCR product; and final extension for 10 min at 72°C. The PCR3 product was gel-purified, digested with *Bam*HI and *Xho*I, and then ligated into the pGEX-6P-1 plasmid. The resultant recombinant plasmid pGEX-SjHSP60 (D24) was transformed into *E. coli* BL21 after confirmation by DNA sequencing. SjHSP60-D24 was prepared in the same manner as SjHSP60.

**Evaluation of liver injury**

To determine hepatocyte damage, serum levels of aspartate aminotransferase (AST) and alanine transaminase (ALT) were assayed by Olympus AU2700 Chemical Analyzer (Olympus, Tokyo, Japan).

**Prediction of protein structure and protein-protein interaction sites**

Using the full-length protein sequence of SjHSP60 as the sole input, the server-based threading program Phyre (<http://www.sbg.bio.ic.ac.uk/phyre>) was used to predict the three-dimensional (3D) structure , which was further analyzed for putative protein-protein interaction sites by using the PredictProtein server ([http://www.predictprotein.org](http://www.predictprotein.org/)) . The crossing angle of the 3D structural model between the helical axes was determined with Rasmol software ([http://www.openrasmol.org](http://www.openrasmol.org/)).

**References**

1. Liu F, Lu J, Hu W, Wang SY, Cui SJ, et al. (2006) New perspectives on host-parasite interplay by comparative transcriptomic and proteomic analyses of Schistosoma japonicum. PLoS Pathog 2: e29.

2. Kelley LA, Sternberg MJ (2009) Protein structure prediction on the Web: a case study using the Phyre server. Nat Protoc 4: 363-371.

3. Rost B, Yachdav G, Liu J (2004) The PredictProtein server. Nucleic Acids Res 32: W321-326.
